# Different contribution of BRINP3 gene in chronic periodontitis and peri-implantitis: a cross-sectional study

**DOI:** 10.1186/s12903-015-0018-6

**Published:** 2015-03-11

**Authors:** Priscila L Casado, Diego P Aguiar, Lucas C Costa, Marcos A Fonseca, Thays CS Vieira, Claudia CK Alvim-Pereira, Fabiano Alvim-Pereira, Kathleen Deeley, José M Granjeiro, Paula C Trevilatto, Alexandre R Vieira

**Affiliations:** Dental School, Clinical Research Unit and Biology Institute, Federal Fluminense University - UFF, Niterói, RJ Brazil; Center of Clinical Research, Orthopedics and Traumatology National Institute – INTO, Rio de Janeiro, RJ Brazil; Department of Dentistry, Federal Fluminense University - UFF, Niterói, RJ Brazil; Department of Biomedicine, Federal Fluminense University- UFF, Niterói, RJ Brazil; Department of Medicine, Health Sciences Center of Lagarto, Federal University of Sergipe (UFS), Lagarto, SE Brazil; Department of Dentistry, Health Sciences Center of Lagarto, Federal University of Sergipe (UFS), Lagarto, SE Brazil; Department of Oral Biology, University of Pittsburgh, Pittsburgh, PA USA; Center for Health and Biological Sciences, Pontifícia Universidade Católica do Paraná (PUCPR), Curitiba, Brazil; National Institute of Metrology (INMETRO), Niterói, RJ Brazil; Department of Oral Biology, School of Dental Medicine, University of Pittsburgh, 3501 Terrace Street, Pittsburgh, PA 15261 USA

**Keywords:** Chronic periodontitis, Peri-implantitis, Genetic polymorphism, Gene expression

## Abstract

**Background:**

Peri-implantitis is a chronic inflammation, resulting in loss of supporting bone around implants. Chronic periodontitis is a risk indicator for implant failure. Both diseases have a common etiology regarding inflammatory destructive response. *BRINP3* gene is associated with aggressive periodontitis. However, is still unclear if chronic periodontitis and peri-implantitis have the same genetic background.

The aim of this work was to investigate the association between *BRINP3* genetic variation (rs1342913 and rs1935881) and expression and susceptibility to both diseases.

**Methods:**

Periodontal and peri-implant examinations were performed in 215 subjects, divided into: healthy (without chronic periodontitis and peri-implantitis, n = 93); diseased (with chronic periodontitis and peri-implantitis, n = 52); chronic periodontitis only (n = 36), and peri-implantitis only (n = 34). A replication sample of 92 subjects who lost implants and 185 subjects successfully treated with implants were tested. DNA was extracted from buccal cells. Two genetic markers of *BRINP3* (rs1342913 and rs1935881) were genotyped using TaqMan chemistry. Chi-square (*p* < 0.05) compared genotype and allele frequency between groups. A subset of subjects (n = 31) had gingival biopsies harvested. The *BRINP3* mRNA levels were studied by C_T_ method (2^ΔΔCT^). Mann–Whitney test correlated the levels of *BRINP3* in each group (*p* < 0.05).

**Results:**

Statistically significant association between *BRINP3* rs1342913 and peri-implantitis was found in both studied groups (p = 0.04). The levels of *BRINP3* mRNA were significantly higher in diseased subjects compared to healthy individuals (p = 0.01).

**Conclusion:**

This study provides evidence that the *BRINP3* polymorphic variant rs1342913 and low level of *BRINP3* expression are associated with peri-implantitis, independently from the presence of chronic periodontitis.

**Electronic supplementary material:**

The online version of this article (doi:10.1186/s12903-015-0018-6) contains supplementary material, which is available to authorized users.

## Background

Peri-implantitis represents a collective term for chronic inflammatory processes affecting the tissues surrounding osseointegrated dental implants and resulting in loss of supporting bone [1]. A series of risk indicators for implant survival, such as lack of oral hygiene, diabetes, smoking, and history of chronic periodontitis [2], has been related to the development of peri-implantitis [3]. Individuals with a history of periodontitis and loss of supporting bone may experience more implant loss and complications around implants than non-periodontitis patients [4]. Periodontal pathogens transmitted from residual dentition to the implant sites can be detected around an implant one month after reopening surgery [5].

Studies concerning the etiopathogenesis of peri-implantitis are based on the development of the periodontal disease. In periodontitis, the local inflammatory reaction to bacterial infection activates the innate immune system, resulting in the release of an array of mediators that propagate the inflammation throughout the gingival tissues [6,7]. Previous studies indicated that chronic periodontitis [8,9], as well as peri-implantitis [10-15], may have a genetic background. However, there is no definitely identified genetic polymorphism that explains biological complications in dental implants [16]. In addition, studies considering the association between genetic predisposition and implant failure have a weak point: the lack of data reporting the periodontal status of the patients.

Carvalho et al. [17] have shown that aggressive periodontitis is associated with markers in *BRINP3* (bone morphogenetic protein/ retinoic acid inducible neural-specific 3, previously referred as *FAM5C*). BRINP3 is related to several cellular functions, such as proliferation, migration, and programmed cell death, and is related to several diseases, including invasion of pituitary tumors, atherosclerosis [18], and myocardial infarction [19]. The *BRINP3* single nucleotide polymorphism rs1935881 was associated with norepinephrine change during exercise [20]; the same *BRINP3* variant we found to be associated to aggressive periodontitis in 389 subjects evaluated from 55 pedigrees [17]. Motivated by the evidence that *BRINP3* contributes to aggressive periodontitis we decided to investigate the role of *BRINP3* in peri-implantitis. Our research group raised two questions: (i) Do *BRINP3* polymorphisms predispose to peri-implantitis in patients with chronic periodontitis? (ii) Does the genetic background associated to *BRINP3* gene predispose to chronic periodontitis and peri-implantitis by the same way? In the present work, we looked for evidence that *BRINP3* may contribute to peri-implantitis and chronic periodontitis by testing for association between genetic markers in *BRINP3* and dental implant failure.

## Methods

### Discovery sample

#### Subject selection

Two hundred and fifteen individuals, presenting 754 osseointegrated endosseous implants, were randomly recruited for the study from the patients’ pool at the Dental Clinics of the Faculty of Dentistry, Fluminense Federal University, Niterói, and Veiga de Almeida University, Rio de Janeiro, Rio de Janeiro, Brazil, during one year. Clinical procedures were conducted according to recommendations from both Universities’ Research Ethics Boards (Registration number 238/10 and 00706212.9.0000.5243 12/09, respectively). Informed consent was obtained from all participants. The baseline clinical parameters for the subject population are shown in Table 1. Subjects answered a personal, medical, and dental history anamnesis (Additional file 1), as well as had their smoking habit and alcohol consumption recorded. The exclusion criterion was implant failure before osseointegration period. The inclusion criteria were: to have at least one osseointegrated endosseous implant, immediate postoperative radiography showing the vertical bone level around implant in order to compare bone level after osseointegration period, periapical radiography showing periodontal status before implant placement, and to be under maintenance care annually for both a clinical and radiographic examination. All implants were placed in a submerged modality (two-stage concept) in sites previously showing favorable bone quality and quantity. Either single crowns or short span fixed partial dentures supported implants.

**Table 1:** **Baseline characteristics of the discovery sample**

	**Healthy (n = 93)**	**Diseased (n = 52)**	**Chronic periodontitis only (n = 36)**	**Peri-implantitis only (n = 34)**	***p*** **-value**					
	**n (%)**	**n (%)**	**n (%)**	**n (%)**	**Healthy**	**Healthy**	**Healthy**	**Diseased**	**Diseased**	**Chronic periodontitis only**
					**X**	**X**	**X**	**X**	**X**	**X**
					**Diseased**	**Chronic periodontitis only**	**Peri-implantitis only**	**Peri-implantitis only**	**Chronic periodontitis only**	**Peri-implantitis only**
Ethnic group										
Whites	83 (89.2)	48 (92.3)	29 (80.5)	29 (85.3)	0.54	0.19	0.54	0.29	0.10	0.59
Non-Whites	10 (10.8)	4 (7.7)	7 (19.5)	5 (14.7)						
Age (years)	51.2 ± 13.3	59.2 ± 10.3	58.5 ± 11.4	55.1 ± 11.8	0.001	0.01	0.68	0.71	1.0	1.0
Sex										
Female	61 (65.6)	38 (73.0)	25 (69.5)	24 (70.6)	0.35	0.67	0.59	0.80	0.71	0.91
Male	32 (34.4)	14 (27.0)	11 (30.5)	10 (29.4)						
Smoking										
Non-smoking	86 (92.5)	45 (86.5)	30 (83.3)	33 (97.0)	0.24	0.12	0.34	0.10	0.67	0.05
Smoking	7 (7.5)	7 (13.5)	6 (16.7)	1 (3.0)						
Alcohol Consumption										
No	49 (52.7)	29 (55.7)	21 (58.3)	24 (70.6)	0.72	0.56	0.07	0.16	0.81	0.28
Yes	44 (47.3)	23 (44.3)	15 (41.7)	10 (29.4)						
Peri-implant Status										
Bleeding on probing	0.12 ± 0.33	0.62 ± 0.48	0.19 ± 0.4	0.55 ± 0.5	0.0001	1.0	0.0001	1.0	0.07	0.002
Spontaneous bleeding	0	0.11 ± 0.32	0	0.14 ± 0.35	0.0001	1.0	0.004	1.0	0.07	0.02
Probing pocket depth (mm)	1.55 ± 0.74	2.4 ± 1.38	1.58 ± 0.66	2.45 ± 1.03	0.04	1.0	0.08	1.0	0.04	0.05
Plaque index	0.03 ± 0.18	0.31 ± 0.46	0.22 ± 0.42	0.17 ± 0.38	0.0001	0.04	0.2	0.4	1.0	1.0
Peri-implant phenotype										
Thin	35 (37.6)	29 (55.7)	18 (50)	13	0.03	0.2	0.95	0.11	0.59	0.32
Thick	58 (62.4)	23 (44.2)	18 (50)	21						
Mobility	0	3	0	2	0.1	1.0	0.3	1.0	0.4	0.6
Platform Type										
External hexagon	44 (47.3)	29 (55.8)	20 (55.5)	21 (61.8)	0.9	1.0	1.0	1.0	1.0	1.0
Internal hexagon	8 (8.6)	6 (11.5)	2 (5.5)	2 (5.9)						
Morse Cone	35 (37.7)	17 (32.7)	13 (36.2)	10 (29.4)						
Others	6 (6.4)		1 (2.8)	1 (2.9)						
Implant region										
Superior	122 (54.5)	126 (50.2)	47 (30.0)	64 (53.3)	0.3	0.0001	0.8	0.5	0.0001	0.0001
Inferior	102 (45.5)	127 (49.8)	110 (70.0)	56 (46.7)						
Osseointegration period (months)	35.53 ± 34.01	37.8 ± 47.1	29.97 ± 21.92	31.7 ± 23.7	1.0	1.0	1.0	1.0	1.0	1.0

Diseased = chronic periodontitis + peri-implantitis.

### Diagnosis of peri-implantitis

All peri-implant regions were clinically and radiographically evaluated. Clinical examination of the peri-implant sites consisted of visual inspection and palpation, analysis of mucosa inflammation, edema, probing pocket depth, bleeding on probing and spontaneous bleeding in four aspects (mesial, buccal, distal and lingual/palatine), plaque index, peri-implant phenotype [21], and implant mobility. One single examiner (P.L.C.) performed all evaluations according to the same guidelines. No calibration protocol was implemented. Conventional periapical radiography, using the paralleling technique, was used to assess the presence of vertical bone loss around the implants by measuring the height of peri-implant bone compared to initial radiography immediate after implant placement. According to the clinical and radiographic characteristics of the peri-implant sites, subjects were characterized as having healthy sites-no clinical signs of inflammation in the peri-implant mucosa and no signs of bone loss–or peri-implantitis–radiographic signs of pathologic bone loss in at least one region (Table 1). Physiological bone loss was characterized considering the normal bone loss of 1 mm at the first year after implant placement and 0.2 mm for subsequent years [22]. According to the implant osseointegration period, the total amount of bone loss was calculated based on the difference between immediate postoperative radiography and diagnosis radiography (at the moment of peri-implant examination). If the total of bone loss was superior to 1 mm and 0.2 mm for year, the patient was considered to have peri-implantitis.

#### Diagnosis of chronic periodontitis

Subjects were characterized as healthy (without history of chronic periodontitis) or with chronic periodontitis (with history of chronic periodontitis).

The diagnosis of chronic periodontitis was established on the basis of radiographic parameters, including periapical radiographic analysis showing horizontal bone resorption before implant placement and assessment of clinical examination and past dental history, in order to differentiate chronic from aggressive periodontitis.

Subjects with chronic periodontitis had to present the following periodontal criteria: (i) diagnosis and classification of generalized chronic periodontitis based on the 1999 Consensus Classification of Periodontal Diseases [23]; (ii) presence of periodontal pockets with clinical attachment loss of ≥5 mm, bleeding on probing, and radiographic bone loss; (iii) age over 35 years; (iv) at least eight teeth with a probing pocket depth of ≥5 mm and bleeding after pocket probing and with at least two of the eight qualifying teeth presenting probing pocket depth ≥ 7 mm by clinical examination or as recorded in the patients chart. All subjects diagnosed with chronic periodontitis had been treated for chronic periodontitis and were under regular maintenance.

Based on peri-implant and periodontal status, subjects were divided into four groups: healthy (without chronic periodontitis history and peri-implantitis, n = 93); diseased (with chronic periodontitis history and peri-implantitis, n = 52); chronic periodontitis only (with chronic periodontitis history without peri-implantitis, n = 36), and peri-implantitis only (with peri-implantitis without chronic periodontitis, n = 34) in order to analyze each disease separately within the discovery sample.

#### DNA collection and purification

Genomic DNA was obtained from saliva samples of 215 subjects following a previously described protocol [24]. The amount and purity of the DNA was determined by spectrophotometer (Nanodrop® 1000, Thermo Scientific, Wilmington, USA). Only DNA samples presenting A260nm/A280nm ratio greater than 1.7 were used.

### Replication sample

#### Subject selection

For replication purposes, we tested a second cohort. A total of 3.578 patient records from the Latin-American Dental Research Institute (ILAPEO) of Curitiba city, Paraná, Brazil, were analyzed in this study. All patients were implant treated (Neodent Implantes Osseointegráveis, Curitiba/PR, Brazil) between 1996 and 2006, and out of 3.578 subjects treated, 126 patients (3.5%) presented implant loss. Early failure associated to peri-implantitis represented the majority of cases (88.2% or 187/212). From these 126 individuals, 92 were evaluated (34 were not evaluated because of death or address change) and diagnosed as peri-implantitis based on previously described criteria. The comparison group was composed of 185 patients treated with osseointegrated implants in function for at least six months and without any loss (healthy peri-implant tissue group). The groups were matched by sex, age, and smoking habits (Table 2). Thus, the sample was composed of 277 unrelated subjects of both sexes with a mean age 53.63 ± 11.14 years (range 27.1–86.9 years). All participants were advised previously about the nature of the study and signed a consent form within a protocol approved by an Institutional Review Board (Ethical Committee in Research at Pontifícia Universidade Católica do Paraná, protocol 323/06). Subjects answered a personal, medical, and dental history anamnesis, as well as had their socioeconomic profile assessed according to Brazilian Economical Classification Criteria–2003; general medical condition, current medication, tooth brushing, use of dental floss and mouthwash, dental appointment frequency, and clinical data-such as number of teeth and placed dental implants–was recorded (Additional file 2). Cases were also subdivided into subjects with single implant loss (n = 69) and cases presenting multiple implant loss (which means more than one implant lost in the same or different sites; n = 23).

**Table 2:** **Baseline characteristics of the replication sample**

	**Healthy peri-implant tissue (n = 185)**	**Peri-implantitis group (n = 92)**	**p-value**
Ethnic Group			
Whites	176 (95.1%)	91 (98.9%)	0.17
Non-Whites	9 (4.9%)	1 (1.1%)	
Mean Age in Years	53.13 ± 11.46	54.63 ± 10.44	0.29
Sex			
Females	122 (66%)	56 (60.9%)	0.43
Males	63 (34%)	36 (39.1%)	
Smoking Status			
Non-Smoking	142 (76.8%)	74 (80.4%)	0.54
Smoking	43 (23.2%)	18 (19.6%)	
Implant Loss			
Single	-	69 (75%)	
Multiple	-	23 (25%)	
Periodontal Status (partially edentulous subjects; n = 236)	**(n = 151)**	**(n = 85)**	
Gingival index	0.64 ± 0.37	0.65 ± 0.53	0.89
Plaque index	0.12 ± 0.23	0.23 ± 0.41	0.96
Calculus index	0.07 ± 0.12	0.13 ± 0.24	0.28
Probing Pocket Depth (mm)	2.72 ± 0.46	2.54 ± 0.47	0.005
Clinical Attachment Loss (mm)	3.62 ± 0.85	3.66 ± 1.07	0.76
Mobility	19	15	0.33

#### Periodontal status

The following parameters were recorded in partially edentulous patients (controls = 151, cases = 85): gingival index [25], plaque index [26], calculus index [27], probing pocket depth, clinical attachment loss, and mobility (absent or present). Periodontal measurements were recorded from four sites on each tooth using a millimeter conventional periodontal probe (PCP11; Hu-Friedy™, Chicago, IL, USA) and all these clinical data were collected by one examiner (F.A.). The periodontal status of all subjects is shown in Table 2.

#### DNA collection and purification

From all subjects, epithelial buccal cells were collected according to a previously described protocol [28]. DNA was extracted from epithelial buccal cells with ammonium acetate 10 M and EDTA 1 mM [29].

### Single nucleotide polymorphism selection and genotyping

We selected two variants in *BRINP3* (rs1342913 and rs1935881) that have been previously associated with aggressive periodontitis [17]. These polymorphisms are located in chromosome 1 at base pair 190121025 in the *BRINP3* intron and at base pair 190066386 in the untranslated region of the gene, respectively. Real time polymerase chain reactions with TaqMan chemistry (Applied Biosystems, Foster City, CA, USA) held in total 1.5 μL/reaction were used for genotyping the selected markers in a PTC-225 tetrad termocycler (Peltier Thermal Cycler, Bio-Rad Life Science, Corston, UK).

### Evaluation of the *BRINP3* expression

Gingival biopsies were harvested from 31 patients from the total dataset of the discovery sample after the osseointegration period, during the implant exposure procedure. Samples were collected from thirteen subjects from the healthy group, nine from the diseased group, six from the chronic periodontitis only group, and three from the peri-implantitis group.

Total RNA from gingival samples was isolated using Trizol® Reagent (Invitrogen™ by Life Technologies, NY, USA) method according to the manufacturer’s protocol. DNase treatment to digest genomic DNA that could lead to false positive gene expression results was done using DNA-free DNase® (Ambion by Invitrogen™ by Life Technologies, NY, USA). RNA integrity was confirmed on a 1.2% agarose denaturing gel electrophoresis stained with SYBR Nucleic Acid Gel Stain® (Invitrogen™ by Life Technologies, NY, USA). RNA quantity was assessed with the spectrophotometer (Nanodrop® 1000, Thermo Scientific, Wilmington, USA). The reverse transcriptase for the synthesis of complementary DNA (cDNA) was made in duplicates, from 1 μg of RNA using the system ImProm-II Reverse Transcription System™ (Promega Corporation, Wisconsin, USA), according to the manufacturer’s protocol. Reactions without transcriptase reverse enzyme were performed. The qPCR reactions were run in the MxPro-Mx3005p software (Stratagene/Agilent Technologies, Wilmington, DE, USA) using the detection system Fast Sybr Green Master Mix (Applied Biosystems, Foster City, CA, USA) with 1.5 μl of cDNA in each reaction. qPCR was performed with an activation step at 95°C for 10 minutes, followed by 40 cycles of denaturation and annealing/extension (95°C for 15 seconds and 60°C for 1 minute). Primers used were for exons 7 and 8 forward 5’ATATACAGGGAGTTTGGCCGC3’ and reverse 5’GAATTCAGGGGCAAGAGGCA3’ and for exons 2 and 3 forward 5’GCCTGCCAAGACAAAGAACC3’ and 5’ CACGAGTGCGTGTCTTCTGA3’. A melt curve was performed for specific amplification analysis. The Livak Method (2^-ΔΔCT^) was used to determine the relative quantification of *BRINP3* expression. Values were normalized with respect to constitutive expression of β-actin (sense 5’-TAC AAT GAG CTG CGT GTG G–3’/ antisense 5’-AGA GGC GTA CAG GGA TAG CA–3’). Data are presented as fold change relative to a calibrator (RNA pool from all samples). All reactions were performed in duplicate.

### Statistical analyses

To access the significance of nominal variables between groups, the chi-square (χ2) test was performed. Continuous variables were expressed as mean and standard deviation. Then, ANOVA or Mann–Whitney tests were used to compare means between groups when the variable was in a normal or non-normal distribution (gene expression). Differences in the prevalence of genotypes and alleles between groups were analyzed using the Pearson chi-square (χ2) test after fitting testing for Hardy-Weinberg equilibrium. Values of *p* < 0.05 were considered statistically significant. Multinominal logistic regression analyses were performed to permit the exploration of many covariates simultaneously. Statistical analyses were performed using STATA 11.1 (StataCorp, Texas USA).

## Results

### Association studies in the discovery sample

Results of the association studies between *BRINP3* markers and peri-implantitis in the discovery sample are summarized in Table 3. Genotype distributions were within Hardy-Weinberg equilibrium (data not shown). The TT genotype of *BRINP3* rs1935881 was associated with peri-implantitis only (p = 0.04). In addition, the frequency of polymorphic genotypes (CT + TT) and T allele was significantly higher in peri-implantitis only cases compared to chronic periodontits only cases (p = 0.009/0.01) and to cases with both peri-implantitis and chronic periodontitis (p = 0.04/0.01). G allele for *BRINP3* rs1342913 also showed high prevalence in peri-implantitis group compared to chronic periodontitis only (p = 0.04).

**Table 3:** **Allele and genotype frequencies of** ***BRINP3*** **markers in the discovery sample**

	**Healthy (n = 93)**	**Diseased (n = 52)**	**Chronic periodontitis only (n = 36)**	**Peri-implantitis only (n = 34)**	***p-value***					
	**n (%)**	**n (%)**	**n (%)**	**n (%)**	**Healthy**	**Healthy**	**Healthy**	**Diseased**	**Diseased**	**Chronic periodontitis only**
					**X**	**X**	**X**	**X**	**X**	**X**
					**Diseased**	**Chronic periodontitis only**	**Peri-implantitis only**	**Peri-implantitis only**	**Chronic periodontitis only**	**Peri-implantitis only**
**rs1342913**										
AA	31 (33.7)	11 (22)	12 (34.3)	7 (21.2)	0.29	0.25	0.34	0.6	0.2	0.08
AG	39 (42.4)	27 (54)	19 (54.3)	15 (45.4)						
GG	22 (23.9)	12 (24)	4 (11.4)	11 (33.4)						
AG + GG	61 (66.3)	39 (78)	23 (65.7)	26 (78.8)	0.14	0.94	0.1	0.93	0.2	0.2
A	101 (54.9)	49 (49)	43 (61.4)	29 (44)	0.34	0.34	0.12	0.5	0.1	0.04
G	83 (45.1)	51 (51)	27 (38.6)	37 (56)						
**rs1935881**										
CC	7 (7.6)	6 (12)	7 (20)	0	0.65	0.1	0.12	0.05	0.4	0.03
CT	40 (43.5)	22 (44)	11 (31.4)	10 (33.3)						
TT	45 (48.9)	22 (44)	17 (48.6)	20 (66.7)						
CT + TT	85 (92.4)	44 (88)	28 (80)	30 (100)	0.38	0.04	0.1	0.04	0.3	0.009
C	54 (29.3)	34 (34)	25 (35.7)	10 (16.6)	0.41	0.32	0.05	0.01	0.8	0.01
T	130 (70.6)	66 (66)	45 (64.3)	50 (83.4)						

Diseased = chronic periodontitis + peri-implantitis.

In order to assess risk factors concurrently, a multiple logistic regression of individual parameters in diseased groups of the discovery sample was performed. This yielded adjusted odds ratios (OR) for individual parameters, including age, sex, ethnic group, smoking, and alcohol consumption (Additional file 3). When analyses were adjusted for other risk factors, no association with *BRINP3* was detected. Age showed a trend for association with individuals with both diseases (p = 0.00001; OR = 3.6, 95% C.I. 0.9-10.7) and chronic periodontitis alone (p = 0.002; OR = 3.0; 95% C.I. 0.8-10.52), suggesting that the difference in the distribution of *BRINP3* alleles between healthy and individuals affected by peri-implantitis is confounded by the difference in the age of both groups (healthy individuals were on average 8 years younger than the affected individuals; Table 1). Also, smoking represented a significant risk factor for individuals with chronic periodontitis only (p = 0.03).

#### Association studies in the replication sample

Taking into consideration the association of *BRINP3* rs1935881 and rs1342913 polymorphisms and the occurrence of peri-implantitis, we analyzed the association between *BRINP3* markers and peri-implantitis in the replication sample based on groups divided according to peri-implant status (Additional file 4). Genotype distributions were within Hardy-Weinberg equilibrium (data not shown). There is an excess of heterozygotes for the *BRINP3* rs1342913 in cases with peri-implantitis (p = 0.04). These results appear to be driven by cases with single dental implant losses (p = 0.027).

#### Gene expression results

**Figure 1:**
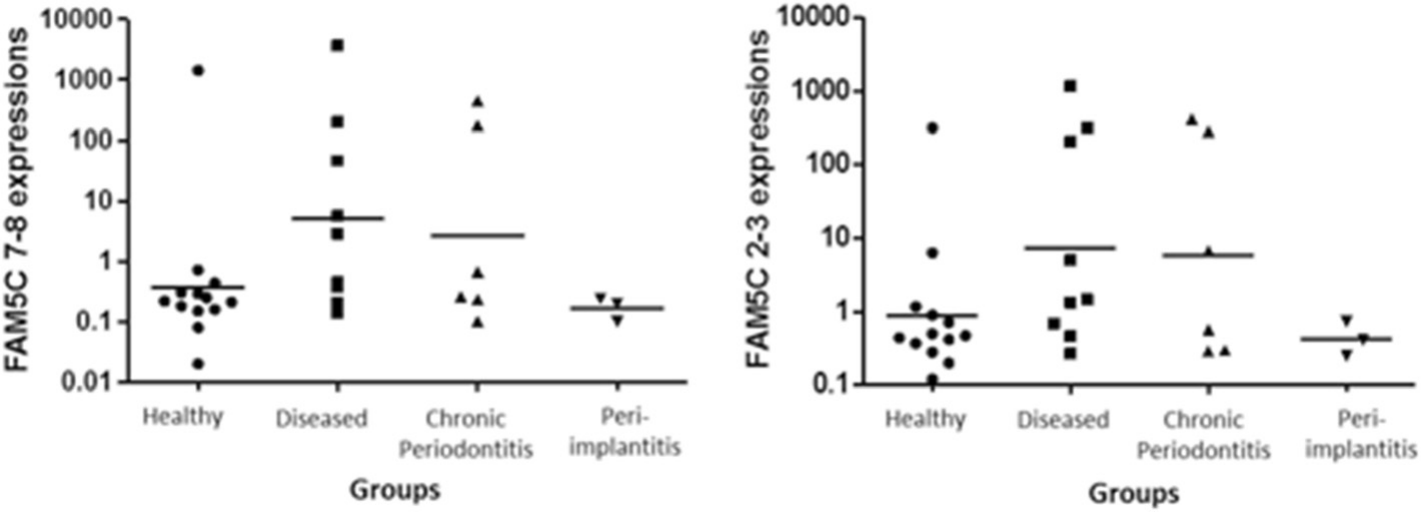
**Expression levels of** ***BRINP3*** **gene (log 10 scale).** Messenger RNA expression levels for *BRINP3* exons 7–8 and 2–3 in gingival samples. Values represent the relative expression values as determined by 2^-∆∆CT^. Diseased = chronic periodontitis + peri-implantitis.

To support the potential association of *BRINP3* genes with the pathogenesis of chronic periodontitis and peri-implantitis, we investigated their expression in diseased and healthy peri-implant tissues (Figure 1). *BRINP3* (exon 7–8 primers) expression was significantly higher in cases with diseased tissues (chronic periodontitis + peri-implantitis) compared to healthy tissues (p = 0.01). We observed a tendency to higher expression in tissues from individuals with both peri-implantitis and chronic periodontitis compared to tissues from individuals with peri-implantitis only (p = 0.05). Although results for *BRINP3* exon 2–3 primers expression were not statistically significant, a similar trend of higher expression in diseased tissues compared to healthy tissues (p = 0.06) and in individuals with both peri-implant disease and chronic periodontitis compared to tissues from individuals with peri-implantitis only (p = 0.07), could be seen. In both *BRINP3* experiments, lower expression was observed in peri-implantitis samples.

## Discussion

Evidence that implant failures tend to increase in subsets of individuals [30] may indicate that specific characteristics of the host, such as genetic factors, can jeopardize the osseointegration process [31]. The previous history of periodontitis has shown to influence the success rate in implant dentistry [2,32] and patients without history of chronic periodontitis present a higher average of implant survival in comparison with individuals with history of periodontitis.^4^ Our work supports the initial hypothesis that peri-implantitis and *BRINP3* are associated and these results appear to be independent from the presence of chronic periodontitis, which suggests that if chronic periodontitis contributes to implant failure, it acts through a pathway not involving *BRINP3*.

The percentage of Black subjects in our sample is smaller that one would expect in the population however this can be explained by the fact that Blacks in Brazil over-represent lower socioeconomic groups and hence are less likely to receive more complex dental treatments such as dental implants. Since the original observation of an association between *BRINP3* and aggressive periodontitis was made in a population data set enriched by Black families, it is possible that the detectable genetic effect of *BRINP3* in dental implant failure is stronger in individuals of African heritage.

In order to elucidate the relation of *BRINP3* as genetic contributor to both diseases, we evaluated the distribution of genotypes of markers which frequencies and linkage disequilibrium relationships were previously described in the International HapMap Project [33]. *BRINP3* is localized in mitochondria and its over-expression leads to increased proliferation and migration of cells [18]. Through complex signaling cascades, mitochondria have the ability to activate multiple pathways that modulate both cell proliferation and, inversely, promote cell arrest and programmed cell death [34] – all phenomena relevant in the pathogenesis of periodontitis and peri-implantitis. Our genetic analysis suggests *BRINP3* polymorphisms are related to the development of peri-implantitis even without chronic periodontitis as a risk factor for peri-implantitis development. Difference in peri-implantitis gene expression was also observed. A tendency to lower *BRINP3* expression in peri-implantitis tissues was clearly identified. In contrast, patients with both diseases showed significant higher *BRINP3* gene expression, which can reflects the potential exacerbated reaction that *BRINP3* can induct in the chronic periodontitis process. It can suggest that the genetic characteristic that conducts resorption reactions around teeth in chronic periodontitis patients probably remains in peri-implant tissue after implant placement, triggering implant failure. However, patients without chronic periodontitis history also can trigger a peri-implant bone loss despite the minimum *BRINP3* mRNA expression. Therefore, these findings still need to be explored.

In spite the fact that chronic periodontitis is a risk indicator for peri-implantitis, previous studies including genetic analysis of peri-implantitis did not differentiate patients with or without chronic periodontitis in order to establish the genetic background of peri-implantitis by itself. Possibly, specific factors are acting by different pathways to originate peri-implantitis in patients without history of chronic periodontitis. Indeed, lower levels of BRINP3 observed in mucosae from peri-implantitis only cases can reflect that peri-implantitis without interaction with chronic periodontitis history has different behaviors.

Based on our results, we can suggest that *BRINP3* genotypes and expression are correlated to peri-implantitis, even without chronic periodontitis history. This association may be particularly relevant in cases of single dental implant losses. However, future studies are necessary, considering the BRINP3 function in vitro and in vivo and its association with bone loss response in separate cases of chronic periodontitis and peri-implantitis.

## Conclusions

This study provides evidence that *BRINP3* polymorphic variants and low level of *BRINP3* expression are associated with peri-implantitis, independently from the presence of chronic periodontitis.

## Additional files

Additional file 1:
**Clinical findings and anamnesis data of the discovery sample.**
Additional file 2:
**Clinical findings and anamnesis data of the replication sample.**
Additional file 3:
**Multinominal logistic regression results for the diseased groups of the discovery samples (reference = healthy individuals).**
Additional file 4:
**Summary of the results of the association between peri-implantitis and** ***BRINP3*** **in the replication sample.**

## Abbreviations

BRINP3: Bone morphogenetic protein/retinoic acid inducible neural-specific 3
FAM5C: Family with sequence similarity 5, member C
DNA: Deoxyribonucleic acid
qPCR: Quantitative polymerase chain reaction
C_T_: Threshold cycle
OR: Odds ratios
C: Celcius
nm: Nanomolars
CI: Confidence intervals
