# Supplementary material for: Different contribution of BRINP3 gene in chronic periodontitis and peri-implantitis: a cross-sectional study
Source: BMC Oral Health. 2015 Mar 11;15:33. doi: 10.1186/s12903-015-0018-6 (PMC4367924; doi:10.1186/s12903-015-0018-6)
Supplement: Additional file 1: — Clinical findings and anamnesis data of the discovery sample. [file 12903_2015_18_MOESM1_ESM.pdf]

Additional File 1. Clinical findings and anamnesis data of the discovery sample.

Parameters	Healthy (n=93) n (%)	Diseased (n=52) n (%)	Chronic Periodontitis Only (n=36) n (%)	Peri-Implantitis Only (n=34) n (%)	*p-value* Healthy X Diseased	Healthy X Chronic Periodontitis Only	Healthy X Peri-Implantitis Only	Diseased X Peri-Implantitis Only	Diseased X Chronic Periodontitis Only	Chronic Periodontitis Only X Peri-Implantitis Only
General Medical Condition										
Systemic disease	24 (25.8)	15 (28.8)	18 (50)	6 (17.6)	0.69	0.008	0.33	0.23	0.04*	0.004
Diabetes	1 (1)	4 (7.7)	1 (2.8)	1 (2.9)	0.03*	0.48	0.44	0.35	0.32	0.96
Rheumatoid diseases	3 (3.2)	2 (3.8)	4 (11)	1 (2.9)	0.84	0.07	0.93	0.82	0.18	0.18
Osteoporosis	1 (1)	0	1 (2.8)	0	0.45	0.48	0.54	-----	0.22	0.32
High blood pressure	12 (12.9)	7 (3.4)	8 (22)	3 (8.8)	0.92	0.18	0.52	0.51	0.28	0.12
Cardiovascular diseases	2 (2.1)	1 (1.9)	1 (2.8)	0	0.92	0.92	0.38	0.41	0.79	0.32
Hypotireoidism	4 (4.3)	1 (1.9)	2 (5.5)	1 (2.9)	0.45	0.76	0.72	0.75	0.35	0.58
Asthma	1 (1)	0	1 (2.8)	0	0.45	0.48	0.54	------	0.22	0.32
Current medication										
Any medication	5 (5.4)	4 (7.7)	4 (11)	1 (2.9)	0.57	0.25	0.56	0.35	0.58	0.18
Antihypertension	11 (11.8)	7 (13.4)	8 (22)	2 (5.9)	0.77	0.13	0.32	0.26	0.28	0.05
Antimicrobials	0	0	0	0	-----	--------	-----	----	-----	----
NSAIDs†	3 (3.2)	2 (3.8)	2 (5.5)	1 (2.9)	0.84	0.53	0.93	0.82	0.70	0.58
SAIDs‡	2 (2.1)	1 (1.9)	2 (5.5)	0	0.93	0.31	0.38	0.41	0.35	0.16
Hormone reposition	4 (4.3)	2 (3.8)	2 (5.5)	1 (2.9)	0.89	0.76	0.72	0.82	0.70	0.58
Antidepressant	4 (4.3)	3 (5.7)	1 (2.8)	1 (2.9)	0.69	0.68	0.72	0.54	0.50	0.96
Clinical measurements										
Edentulism	12 (12.9)	13 (25)	12 (33.3)	4 (11.8)	0.06	0.007	0.86	0.13	0.39	0.03
Caries Experience (DMFT*)	17.72±8.11	24.8±6.38	24.16±7.97	20.58±8.03	0.0001	0.0001	0.4	0.07	1.0	0.3
Dental anomalies	3 (3.2)	0	0	0	0.3	0.27	0.218	-----	----	----
Dental agenesis	3 (3.2)	0	0	2 (5.9)	0.3	0.27	0.28	0.08	-----	0.13
Periodontal phenotype										
Thin#	12 (12.9)	29 (55.7)	17 (47.2)	14 (41.2)	0.000001	0.00002	0.0004	0.18	0.42	0.61
Thick	81 (87.1)	23 (44.3)	19 (52.8)	20 (58.8)						

Diseased= chronic periodontitis + peri-implantitis; # Chi-squared test; † Non-steroidal anti-inflammatory drugs; ‡ Steroidal anti-inflammatory drugs; *DMFT= Decayed, Missing due to caries, Filled Teeth; #presence of apertures in the subjacent bone and friable gingiva.
