# Supplementary material for: Different contribution of BRINP3 gene in chronic periodontitis and peri-implantitis: a cross-sectional study
Source: BMC Oral Health. 2015 Mar 11;15:33. doi: 10.1186/s12903-015-0018-6 (PMC4367924; doi:10.1186/s12903-015-0018-6)
Supplement: Additional file 2: — Clinical findings and anamnesis data of the replication sample. [file 12903_2015_18_MOESM2_ESM.pdf]

Additional File 2. Clinical findings and anamnesis data of the replication sample.

	Successfully Dental Implant Treated Controls (n=185)		Dental Implant Loss Cases (n=92)		p-value
	n	%	n	%	
Social Profile					
A1/A2/B1	95	51.35	46	50	0.9*
B2/C/D	90	48.65	46	50	
Medical treatment					
Yes	75	40.54	50	54.35	0.04*
No	110	59.46	42	45.65	
General medical condition					
Systemic disease	127	64.65	68	73.91	0.4*
Diabetes	09	4.86	02	2.17	0.34*
Rheumatoid diseases	34	18.38	25	27.17	0.12*
Osteoporosis	03	1.62	02	2.17	0.99*
High blood pressure	35	18.92	24	26.09	0.21*
Cardiovascular diseases	10	5.4	08	8.7	0.31*
Hypotireoidism	18	9.73	10	10.87	0.83*
Current medication					
Any medication	46	24.86	17	18.48	0.29*
Antihypertension	30	16.22	21	22.83	0.19*
Antimicrobials	15	8.11	07	7.61	1.0*
NSAID†	06	3.24	06	6.52	0.22*
SAID‡	04	2.16	03	3.26	0.69*
Hormone reposition	33	17.84	12	13.04	0.39*
Brushing daily					
One time	09	4.86	06	6.52	0.71§
Two times	42	22.7	16	17.39	
Three times	110	59.46	56	60.87	
More than three times	24	12.97	14	15.22	
Dental floss daily					
Yes	122	65.95	61	66.3	0.34§
No	37	20	23	25	
Infrequent	26	14.05	08	8.7	
Mouth washing daily					
Yes	60	42.43	29	31.52	0.77§
No	85	45.95	46	50	
Infrequent	40	21.62	17	18.48	
Clinical appointments ¶		6.42 ± 4.94		6.28 ± 4.59	0.85
Clinical measurements					
Edentulism	34	18.38	07	7.61	0.02*
Present teeth ¶		16.59 ± 9.76		17.05 ± 8.31	0.88
Placed implants ¶		4.44 ± 3.12		5.82 ± 3.65	0.001

*Fisher's exact test; †Non-steroidal anti-inflammatory drugs; ‡Steroidal anti-inflammatory drugs; §Chi-square test; ¶Mean ± standard deviation.
