# Supplementary material for: Different contribution of BRINP3 gene in chronic periodontitis and peri-implantitis: a cross-sectional study
Source: BMC Oral Health. 2015 Mar 11;15:33. doi: 10.1186/s12903-015-0018-6 (PMC4367924; doi:10.1186/s12903-015-0018-6)
Supplement: Additional file 3: — Multinominal logistic regression results for the diseased groups of the discovery samples (reference = healthy individuals). [file 12903_2015_18_MOESM3_ESM.pdf]

Additional File 3. Multinominal logistic regression results for the diseased groups of the discovery samples (reference = healthy individuals).

Groups	*BRINP3* rs1342913			*BRINP3* rs1935881			Age	Sex	Ethnic Group	Smoking	Alcohol
	AA	AG	GG	CC	CT	TT					
Diseased											
p-value	1.0	0.19	0.84	1.0	0.61	0.32	0.00001	0.14	0.85	0.14	0.92
Odds Ratio (95% Confidence Interval)	1.0	1.86 (0.75-4.58)	1.09 (0.37-3.25)	1.0	1.01 (0.44-2.3)	1.81 (0.46-7.12)	3.6 (0.90-10.7)	0.53 (0.23-1.23)	0.88 (0.24-3.18)	2.63 (0.71-9.64)	1.04 (0.47-2.30)
Chronic Periodontitis Only											
p-value	1.0	0.45	0.16	1.0	0.39	0.11	0.002	0.39	0.11	0.03	0.33
Odds Ratio (95% Confidence Interval)	1.0	1.7 (0.64-4.48)	0.39 (0.09-1.57)	1.0	0.88 (0.26-1.82)	3.0 (0.74-12.11)	3.0 (0.80-10.52)	0.66 (0.26-1.69)	1.2 (0.67-2.19)	4.45 (1.12-17.61)	0.63 (0.25-1.59)
Peri-Implantitis Only											
p-value	1.0	0.5	0.49	1.0	0.41	0.98	0.14	0.76	0.7	0.57	0.29
Odds Ratio (95% Confidence Interval)	1.0	1.34 (0.47-3.76)	1.38 (0.43-4.4)	1.0	0.7 (0.26-1.59)	8.24 (0-22.3)	1.03 (0.99-1.07)	0.87 (0.34-2.17)	1.26 (0.37-4.25)	0.52 (0.05-4.89)	0.61 (0.24-1.52)

Diseased= chronic periodontitis + peri-implant disease.
