# Supplementary material for: Different contribution of BRINP3 gene in chronic periodontitis and peri-implantitis: a cross-sectional study
Source: BMC Oral Health. 2015 Mar 11;15:33. doi: 10.1186/s12903-015-0018-6 (PMC4367924; doi:10.1186/s12903-015-0018-6)
Supplement: Additional file 4: — Summary of the results of the association between peri-implantitis and BRINP3 in the replication sample. [file 12903_2015_18_MOESM4_ESM.pdf]

Additional File 4. Summary of the results of the association between peri-implantitis and *BRINP3* in the replication sample.

Genotypes	*BRINP3* rs1342913		*BRINP3* rs1935881	
	Peri-implantitis Group (n=92) n (%)	Healthy Peri-implant Tissue (n=185) n (%)	Peri-implantitis Group (n=92) n (%)	Healthy Peri-implant Tissue (n=185) n (%)
	All Losses (n=92)	Controls	All Losses (n=92)	Controls
AA	22 (25.0)	53 (29.0)	7 (8.1)	14 (7.7)
AB	49 (55.7)	74 (40.4)	35 (40.7)	85 (47.0)
BB	17 (19.3)	56 (30.6)	44 (51.2)	82 (45.3)
p-value	0.045		0.62	
	Single Losses (n=69)	Controls	Single Losses (n=69)	Controls
AA	13 (23.6)	53 (29.0)	6 (11.1)	14 (7.7)
AB	33 (60.0)	74 (40.4)	23 (42.6)	85 (47.0)
BB	9 (16.4)	56 (30.6)	25 (46.3)	82 (45.3)
p-value	0.027		0.69	
	Multiple Losses (n=23)	Controls	Multiple Losses (n=23)	Controls
AA	9 (27.3)	53 (29.0)	1 (3.1)	14 (7.7)
AB	16 (48.5)	74 (40.4)	12 (37.5)	85 (47.0)
BB	8 (24.2)	56 (30.6)	19 (59.4)	82 (45.3)
p-value	0.65		0.29	

Note: Differences in the total number of genotypes and the total sample are due to PCR failure.
